# Prothrombin complex concentrates and a specific antidote to dabigatran are effective *ex-vivo* in reversing the effects of dabigatran in an anticoagulation/liver trauma experimental model

**DOI:** 10.1186/cc13717

**Published:** 2014-02-05

**Authors:** Oliver Grottke, Joanne van Ryn, Henri MH Spronk, Rolf Rossaint

**Affiliations:** 1 Department of Anaesthesiology, RWTH Aachen University Hospital, Pauwelsstrasse 30, D-52074 Aachen, Germany; 2 CardioMetabolic Diseases Research, Boehringer Ingelheim GmbH & Co. KG, Birkendorfer Str 65, D-88397 Biberach, Germany; 3 Laboratory for Clinical Thrombosis and Haemostasis, Department of Internal Medicine, Cardiovascular Research Institute Maastricht, Maastricht University Medical Center, 6200 MD Maastricht, The Netherlands

## Abstract

**Introduction:**

New oral anticoagulants are effective alternatives to warfarin. However, no specific reversal agents are available for life-threatening bleeding or emergency surgery. Using a porcine model of trauma, this study assessed the ability of prothrombin complex concentrate (PCC), activated PCC (aPCC), recombinant FVIIa (rFVIIa) and a specific antidote to dabigatran (aDabi-Fab) to reverse the anticoagulant effects of dabigatran.

**Methods:**

Dabigatran etexilate (DE) was given orally for 3 days (30 mg/kg bid) and intravenously on day 4 to achieve consistent, supratherapeutic concentrations of dabigatran. Blood samples were collected at baseline, after oral DE, after intravenous dabigatran, and 60 minutes post-injury. PCC (30 and 60 U/kg), aPCC (30 and 60 U/kg), rFVIIa (90 and 180 μg/kg) and antidote (60 and 120 mg/kg) were added to blood samples *ex-vivo*. Coagulation was assessed by thromboelastometry, global coagulation assays and diluted thrombin time.

**Results:**

Plasma concentrations of dabigatran were 380 ± 106 ng/ml and 1423 ± 432 ng/ml after oral and intravenous administration, respectively, and all coagulation parameters were affected by dabigatran. Both PCCs and aDabi-Fab, but not rFVIIa, reversed the effects of dabigatran on thromboelastometry parameters and prothrombin time. In contrast, aPTT was only normalised by aDabi-Fab. Plasma concentration (activity) of dabigatran remained elevated after PCC and rFVIIa therapy, but was not measureable after aDabi-Fab.

**Conclusion:**

In conclusion, PCC and aPCC were effective in reducing the anticoagulant effects of dabigatran under different conditions, while aDabi-Fab fully corrected all coagulation measures and decreased the plasma concentration of dabigatran below the limit of detection. No significant effects were observed with rFVIIa.

## Introduction

Uncontrolled post-traumatic bleeding is the leading cause of potentially preventable death among trauma patients [1]. Early coagulation abnormalities are frequent in severely injured trauma patients and are associated with substantially increased mortality [2]. Patients’ tendency towards perioperative and trauma-induced bleeding is further increased by anticoagulant medication. The direct oral anticoagulant (DOAC) dabigatran, a direct thrombin inhibitor, is administered orally as the pro-drug dabigatran etexilate (DE) and is characterised by a rapid onset of action, few drug or food interactions, no requirement for routine coagulation monitoring and a short half-life of 12 to 14 hours [3].

Dabigatran is approved in many countries for the primary prevention of venous thromboembolic events in adult patients who have undergone elective total hip or total knee replacement surgery, and/or for the prevention of stroke and systemic embolism in adult patients with non-valvular atrial fibrillation [3,4]. Dabigatran directly inhibits both free and clot-bound thrombin, and this impedes the conversion of fibrinogen to fibrin, thus preventing thrombus development. Results from a large multicentre randomised controlled trial including 18,113 patients with atrial fibrillation (RE-LY) suggest a low overall risk of bleeding complications [5]. Because of this and the fact that clinical use of DOACs began relatively recently, experience in the management of bleeding complications associated with DOACs is limited. However, in the case of life-threatening bleeding following injury, immediate and prompt reversal of anticoagulation is required [6]. At the present time, no specific antidotes are licensed for reversal of the anticoagulant effects of dabigatran.

Experimental data show that dabigatran may be removed from the circulation by dialysis [7]. This approach is reported to be effective in patients with end-stage renal disease and several case reports have been published showing that dialysis is effective in reducing plasma concentrations of dabigatran [8-10]. However, this procedure may not be feasible in haemodynamically unstable patients with haemorrhagic shock. Prothrombin complex concentrate (PCC), activated PCC (aPCC) and recombinant activated factor VII (rFVIIa) have been proposed as candidates for reversing the anticoagulant effects of dabigatran. However, results from initial experimental trials are inconclusive and they do not account for different dabigatran concentrations or the combined effects of dabigatran and severe injury such as trauma [11-13]. Data from the few studies that have been performed in humans are also inconclusive. One study of PCC in healthy volunteers previously receiving dabigatran showed an increase in endogenous thrombin potential [14], whereas in another study PCC did not reverse the effect of dabigatran as measured by activated partial thromboplastin time (aPTT) [15]. A specific antibody fragment to dabigatran (aDabi-Fab) is in development, and in a rat model of anticoagulation it rapidly reversed the anticoagulant activity of dabigatran [16]. However, this antidote is not yet licensed for clinical use.

This porcine study was performed to evaluate the potential use of commonly available haemostatic agents (PCC, aPCC and rFVIIa), as well as the specific antidote aDabi-Fab, to reverse dabigatran-induced coagulopathy in an anticoagulation/trauma model. In addition, the study investigated the sensitivity of different coagulation tests, including thromboelastometry variables, for diagnosis and reversal of dabigatran/trauma-induced coagulopathy.

## Materials and methods

### Ethics and anaesthesia

All experiments were performed in accordance with German legislation governing animal studies following the *Principles of Laboratory Animal Care*. Ethical approval for these studies was obtained from the regional governmental animal care and use office (No. 84–02.04.2012.A197). Before surgery, pigs were housed in ventilated rooms and allowed to acclimatise to their surroundings for a minimum of seven days. Animals were fasted overnight before surgical procedures, with unrestricted access to water.

Prior to surgery, DE (Pradaxa, Boehringer Ingelheim, Biberach, Germany) was administered orally twice daily for 3 days (30 mg/kg *bid*). On the day of surgery, animals received an intramuscular injection of 4 mg/kg azaperone (Stresnil, Janssen, Neuss, Germany) and 0.1 mg/kg atropine (atropine sulphate, B Braun, Melsungen, Germany) as premedication. Anaesthesia was induced by intravenous injection of 3 mg/kg propofol (Disoprivan, Astra Zeneca, Wedel, Germany) followed by orotracheal intubation. The animals were ventilated with a tidal volume of 8 mL/kg and 16 to 22 breaths/minute (Cato, Draeger, Luebeck, Germany) to maintain end-tidal carbon dioxide between 36 and 42 mmHg. Anaesthesia was maintained with isoflurane (Forane, Abbott Laboratories Inc., Abbott Park, IL, USA) at an end-tidal concentration of 1% and a continuous infusion of fentanyl (Janssen, Neuss, Germany) at 3 to 4 μg/kg/h. Ringer’s solution (Sterofundin, Braun, Germany) was infused at 4 mL/kg/h initially, increasing to 10 mL/kg/h after laparotomy until infliction of trauma. Throughout the experiment, body temperature was maintained at 36.5 to 37.0°C with a warming blanket.

Monitoring included electrocardiography (ECG), tail pulse oximetry, temperature, and arterial and central venous pressure measured by femoral catheters connected to a standard anaesthesia monitor (AS/3, Datex Ohmeda, Helsinki, Finland).

### Surgical preparation and dabigatran infusion

Two 8.5-Fr catheters were surgically implanted in the right and left jugular veins for volume substitution and insertion of a pulmonary artery catheter. The right femoral artery was cannulated with an 18-G catheter to collect blood samples and to measure continuous arterial pressure. After line placement, a midline laparotomy with cystostomy was performed. Subsequently, dabigatran (active substance; Boehringer Ingelheim, Biberach, Germany) was infused at a rate of 0.77 mg/kg/h for 30 minutes and 0.52 mg/kg/h for 60 minutes to achieve consistent, supratherapeutic plasma concentrations of dabigatran.

A reproducible blunt liver injury was induced by clamping once through the parenchyma of the right middle liver lobe, using a custom-made instrument; the procedure has been described previously by our group [17]. Five minutes after injury and following haemorrhagic shock, all animals received a fluid bolus of 35 mL/kg of Ringer’s solution followed by continuous infusion of 40 mL/kg/h until four hours post-injury. Sixty minutes after trauma, the abdomen was reopened and the blood was collected to determine the total blood loss post-injury.

### Haemostatic agents

The following haemostatic agents were tested: PCC (Beriplex P/N 250 (US brand-name Kcentra), CSL Behring GmbH, Marburg, Germany; lot 56560111C), aPCC (FEIBA, Baxter, Vienna, Austria; lot VNP5L003), and rFVIIa (NovoSeven, NovoNordisk, Denmark; lot LR04350). In addition, a specific antibody fragment to dabigatran being developed as an antidote (aDabi-Fab, Boehringer Ingelheim, Biberach, Germany; lot 6001325) was also tested [16]. PCC, aPCC and rFVIIa were reconstituted with sterile water according to the manufacturer’s instructions immediately prior to administration. The aDabi-Fab was obtained in Tween 20 buffer (25 mM acetate, 220 mM sorbitol and 0.2% polysorbate 20) at a concentration of 44 mg/mL. Aliquots were stored at −80°C and thawed at 37°C for 10 minutes prior to application.

### Blood collection and *ex vivo* addition of haemostatic agents

Blood samples were collected into sodium citrate (Sarsted, Nuembrecht, Germany) at the following four time points: baseline (3 days before oral administration of dabigatran was started), 12 h after the last oral dose of DE, which represents trough levels of dabigatran (low dabigatran level), after the 90-minute dabigatran infusion, which represents peak levels of dabigatran (high dabigatran level) and 60 minutes post-injury (post-trauma), which was also 60 minutes after stopping the dabigatran infusion and induction of blunt trauma injury. Placebo (saline), PCC, aPCC, rFVIIa or aDabi-Fab was added *ex vivo* to each citrated whole blood sample from each time point. The concentration of PCC and aPCC added was equivalent to the plasma concentrations achieved with 30 U/kg and 60 U/kg; rFVIIa was similarly added to achieve plasma levels equivalent to those achieved with 90 μg/kg and 180 μg/kg. aDabi-Fab was added at a concentration to achieve plasma levels equivalent to 30 or 60 mg/kg.

### Analytical methods including coagulation assays and thromboelastometry

Haemoglobin (Hb) concentrations were measured with a blood gas analyser (ABL500, Radiometer, Copenhagen, Denmark). Prothrombin time (PT, Innovin), aPTT (Actin FS) and fibrinogen concentration (thrombin reagent) were determined by standard laboratory methods using the appropriate tests (all from Dade Behring, Marburg, Germany) on a coagulometer (MC 4 plus, Merlin Medical, Lemgo, Germany). Dabigatran plasma concentration was determined using the diluted thrombin time (Hemoclot, HyphenBiomed, Neuville sur-Oise, France).

Coagulation was assessed in whole blood using a thromboelastometry device (ROTEM, Tem International GmbH, Munich, Germany) and the EXTEM assay. The following parameters were measured: clotting time (CT, s), clot formation time (CFT, s) and maximum clot firmness (MCF, mm).

### Statistical analysis

Statistical analysis was performed using PASW 18 (SPSS, Chicago, IL, USA). For graphical purposes, GraphPad Prism (Version 6.0, GraphPad Software, Inc., La Jolla, CA, USA) was used. Differences between the control and intervention groups were analysed with a one-way analysis of variance (ANOVA), with the Dunnett *post hoc* test for multiple comparisons. ‘Non-measurable’ was entered for clot formation time (CFT) when the required clot amplitude of 20 mm was not reached within 4,000 seconds. Data are presented as mean ± SD. Statistical tests were performed two-tailed and *P*-values <0.05 were considered statistically significant.

## Results

Five male German land-race pigs were included in this *ex vivo* study; the animals’ bodyweights ranged between 37 and 42 kg.

### Effects of oral administration of DE and intravenous infusion of dabigatran

**Figure 1:**
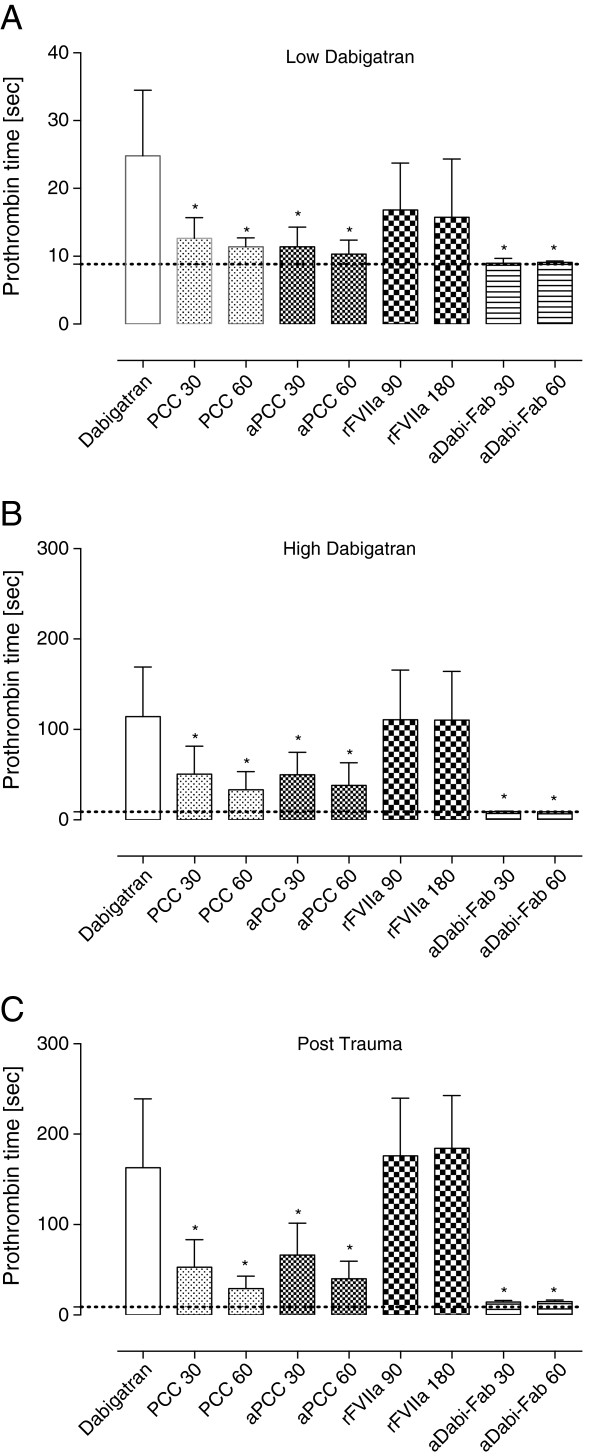
**Prothrombin time at the low dabigatran (A), high dabigatran (B) and post-trauma (C) time points.** Prothrombin time was obtained on a coagulometer using Innovin to determine the effect of *ex vivo* haemostatic therapy at various time points. Grey dotted lines indicate mean baseline values. ^*^P <0.05 versus control. PCC, prothrombin complex concentrate; aPCC, activated PCC; rFVIIa, recombinant activated factor VII; aDabi-Fab, antibody fragment to dabigatran.

**Figure 2:**
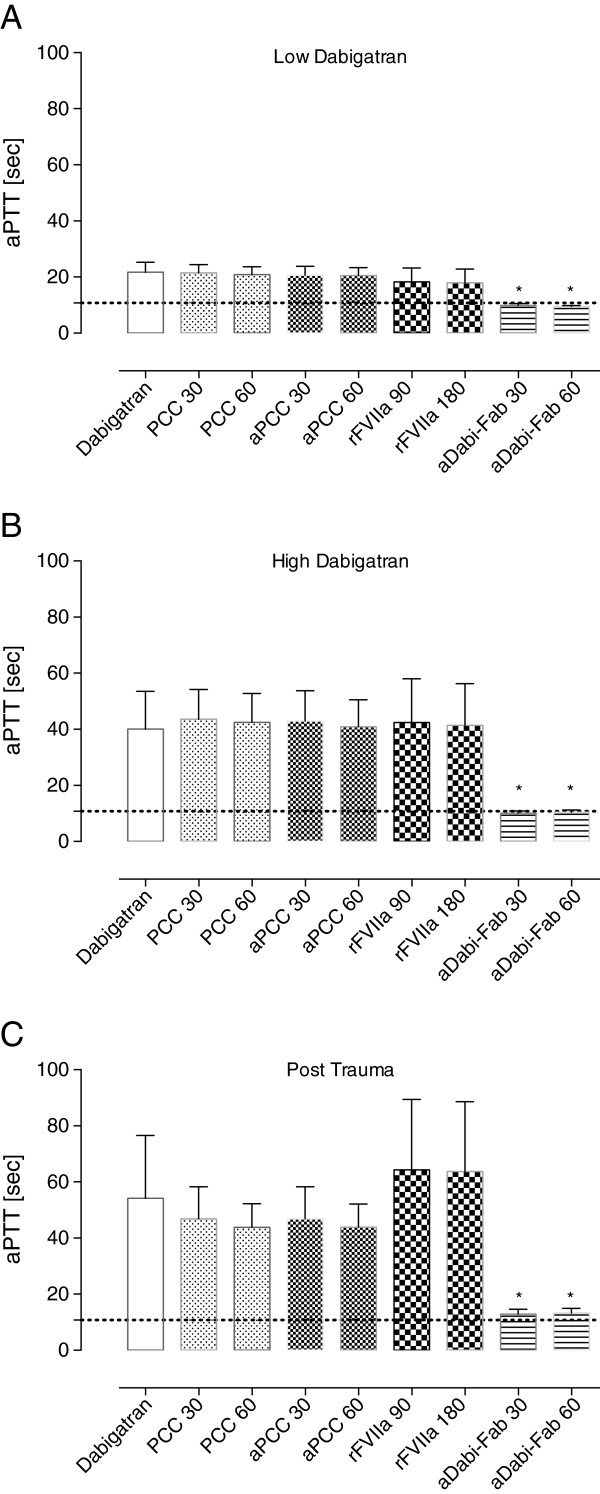
**aPTT at the low dabigatran (A), high dabigatran (B) and post-trauma (C) time-points.** Activated partial thromboplastin time (aPTT) was obtained on a coagulometer to determine the effect of hemostatic therapy at various time points. Grey dotted lines indicate mean baseline values. Data are shown as mean ± SD. ^*^P <0.05 versus control. PCC, prothrombin complex concentrate; aPCC, activated PCC; rFVIIa, recombinant activated factor VII; aDabi-Fab, antibody fragment to dabigatran.

**Figure 3:**
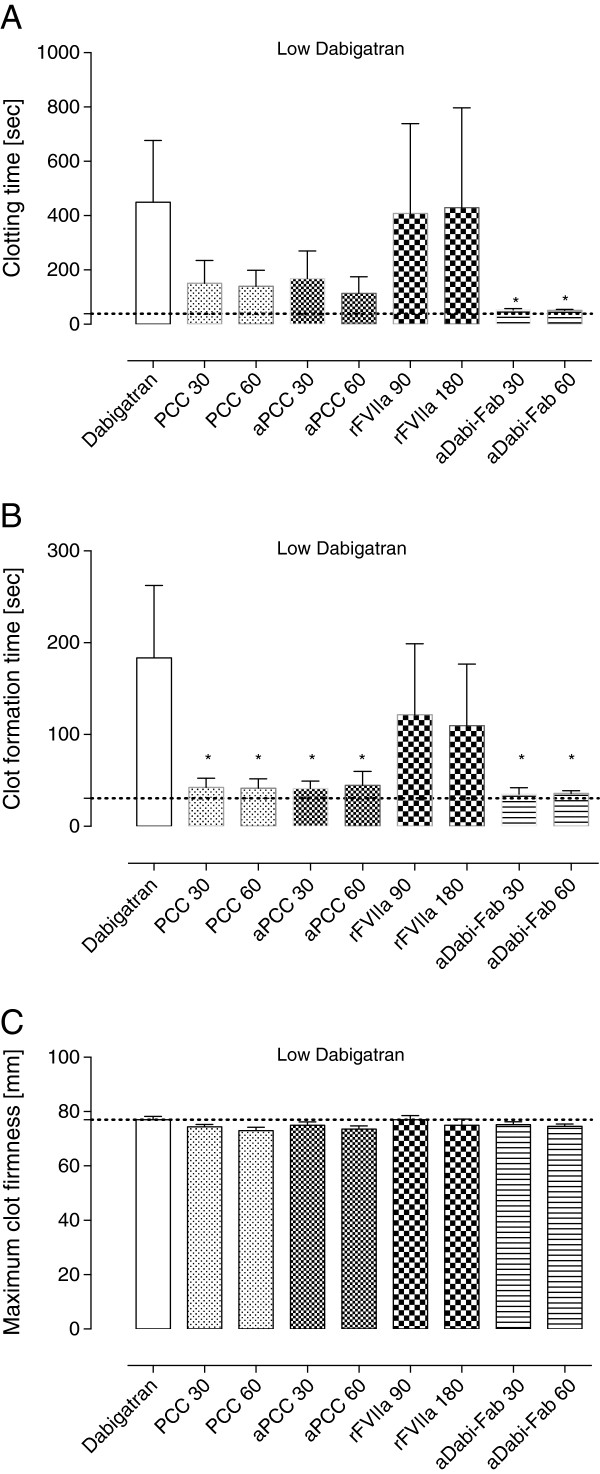
**Thromboelastometry parameters after oral administration of dabigatran etexilate.** The ROTEM coagulation analyzer (TEM international, Munich, Germany) was used for thromboelastometry. For activation the ExTEM reagent containing tissue factor as starting reagent was used according to the manufacturer instructions. **(A)** The clotting time (CT) represents the initiation of clot formation and corresponds to the reaction time. **(B)** clot formation time (CFT) reflects the coagulation time until 20 mm of amplitude are reached. **(C)** The maximum clot firmness (MCF) reflects the strength of a resulting clot. Data are shown as mean ± SD. ^*^P <0.05 versus control. PCC, prothrombin complex concentrate; aPCC, activated PCC; rFVIIa, recombinant activated factor VII; aDabi-Fab, antibody fragment to dabigatran.

All coagulation parameters were within reference ranges at baseline (grey dotted line in all figures). After three days of oral DE, the mean plasma concentration of dabigatran was 380 ± 106 ng/mL (low dabigatran, in Table 1). Laboratory coagulation parameters were prolonged compared with baseline: PT from 9 ± 1 to 25 ± 8 s and aPTT from 13 ± 1 to 22 ± 4 s (control, Figures 1A and 2A). Accordingly, the EXTEM variables CT and CFT were also substantially prolonged (control, Figure 3A and B). However, no effects of oral DE administration on clot strength (MCF) or concentration of haemoglobin, platelets or fibrinogen were observed (control, Figure 3C and Table 2).

**Table 1:** Plasma concentration (activity, measured by diluted thrombin time) of dabigatran (ng/mL) during the study

	**Low dabigatran**	**High dabigatran**	**Post-trauma**
Control	380 ± 106	1423 ± 432	1021 ± 238
PCC (30; 60 U/kg)	299 ± 101; 302 ±115	1276 ± 443; 1273 ± 479	814 ± 81; 801 ± 185
aPCC (30; 60 U/kg)	291 ± 108; 288 ± 107	1199 ± 452; 1188 ±449	785 ± 166; 795 ± 159
rFVIIa (90; 180 μg/kg)	270 ± 86; 267 ± 87	1266 ± 500; 1295 ± 496	766 ± 194; 797 ± 161
aDabi-Fab (30; 60 mg/kg)	0.0 ± 0.0; 0.0 ± 0.0	0.0 ± 0.0; 0.0 ± 0.0	0.0 ± 0.0; 0.0 ± 0.0

Because dabigatran etexilate had not been administered, plasma concentrations of dabigatran were not measured at baseline.

Data are shown as mean ± SD. PCC, prothrombin complex concentrate; aPCC, activated PCC; rFVIIa, recombinant activated factor VII; aDabi-Fab, antibody fragment to dabigatran.

**Table 2:** Haematological parameters and fibrinogen concentration during the study

	**Baseline**	**Low dabigatran**	**High dabigatran**	**Post-trauma**
Haemoglobin (g/L)	10.5 ± 0.7	10.2 ± 0.8	9.5 ± 0.4	4.5 ± 0.7
Platelets (× 10^3^/μL)	388 ± 51	403 ± 36	355 ± 66	202 ± 50
Fibrinogen (mg/dL)	161 ± 16	135 ± 13	118 ± 19	64 ± 12

Data are shown as mean ± SD.

**Figure 4:**
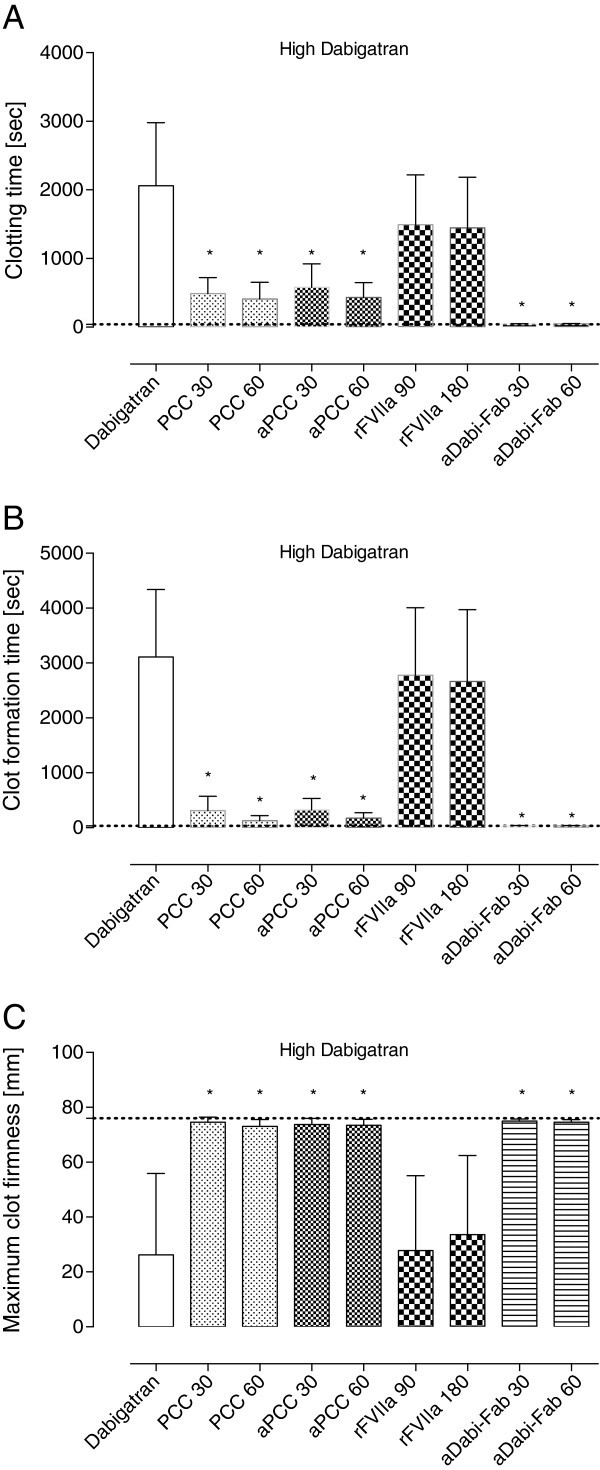
**Thromboelastometry parameters after intravenous administration of dabigatran.** After 90 minutes of intravenous infusion of dabigatran, the EXTEM assay was performed using a ROTEM device. Data are shown as mean ± SD. ^*^P <0.05 versus control. PCC, prothrombin complex concentrate; aPCC, activated PCC; rFVIIa, recombinant activated factor VII; aDabi-Fab, antibody fragment to dabigatran.

**Figure 5:**
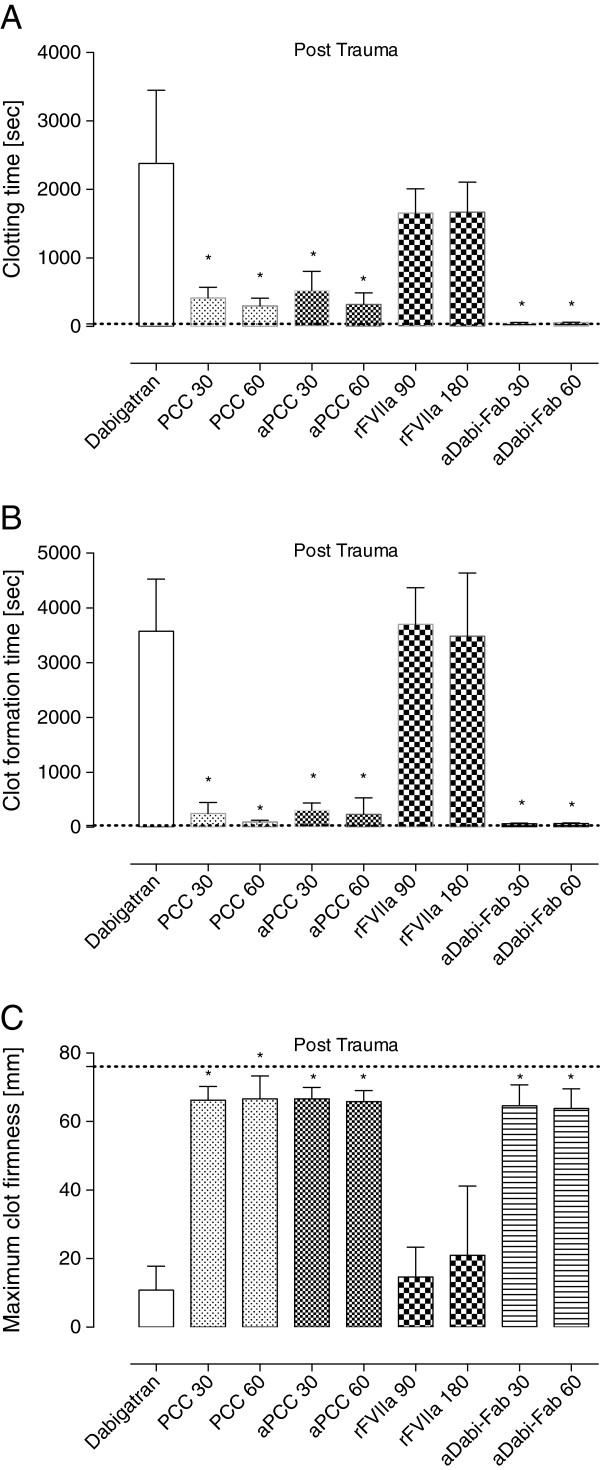
**Thromboelastometry parameters 60 minutes after trauma.** Sixty minutes after stopping the dabigatran infusion and the infliction of blunt liver injury trauma, a ROTEM device was used to measure the impact of dabigatran reversal. Data are shown as the mean ± SD. ^*^P <0.05 versus control. PCC, prothrombin complex concentrate; aPCC, activated PCC; rFVIIa, recombinant activated factor VII; aDabi-Fab, antibody fragment to dabigatran.

Following the 90-minute infusion of dabigatran, the mean plasma concentration (activity) of dabigatran increased to 1423 ± 432 ng/mL. This supratherapeutic level was associated with a further prolongation of PT, aPTT, and the EXTEM variables CT and CFT (Figures 1B, 2B and 4). These changes in coagulation parameters were compounded by blood loss following trauma (total blood loss at 60 minutes 1978 ± 265 mL) and dilution following the infusion of crystalloids. Sixty minutes after trauma, four out of five animals had no measurable clot formation (EXTEM CFT ≥4,000 s), and clot strength (EXTEM MCF) had reduced to 11 ± 7 mm (Figure 5). At the same time, plasma fibrinogen concentration had decreased to 64 ± 12 mg/dL and the mean haemoglobin level had dropped to 4.5 ± 0.6 g/L (Table 2). In addition, further prolongation of PT and aPTT was seen (control, Figures 1C and 2C).

### Measurements after haemostatic therapy *ex vivo*

#### Ex-vivo treatment with PCC

The effects of dabigatran anticoagulation, alone (after both oral and intravenous administration) and compounded by trauma-induced coagulopathy, were reduced by PCC, as shown by significant decreases versus control in PT, CT and CFT (Figures 1, 3, 4 and 5). Increased effects were apparent with the higher concentration of PCC after intravenous administration of dabigatran (high plasma concentration of dabigatran) and, in addition to the effects on PT, CT and CFT, clot strength (EXTEM MCF) almost returned to baseline with both doses of PCC. A similar pattern was observed following trauma: PT, CT and CFT were significantly shortened by PCC, and clot strength was returned to levels close to baseline (Figures 1C and 5A-C). Despite approximately 80 to 90% reversal of the effects on these parameters, PCC treatment had no effect on aPTT at any time point (Figure 2A-C), and the plasma level (activity, measured by diluted thrombin time) of dabigatran was also unchanged by PCC.

#### Ex vivo treatment with aPCC

Dabigatran-induced coagulopathy, both after oral intake and infusion, was reversed by aPCC application, with very similar response patterns to those observed with PCC (Figures 1, 3, 4 and 5). Decreases in PT, CT and CFT were observed, and MCF returned close to baseline after intravenous dabigatran and trauma, although, as with PCC these parameters were not fully restored to baseline levels. Also as with PCC, aPCC had no significant effect on aPTT or levels of dabigatran.

#### Ex vivo treatment with rFVIIa

*Ex-vivo* addition of rFVIIa had no significant effect on any of the coagulation parameters at any time point. This was true for both doses of rFVIIa. In addition, rFVIIa had no impact on the plasma concentration of dabigatran at any time point.

#### Ex vivo treatment with aDabi-Fab

After oral DE and intravenous dabigatran, both concentrations of aDabi-Fab restored coagulation parameters to their baseline values or similar. Even after trauma, PT, aPTT, CT, and CFT were reversed to baseline values (Figures 1C, 2C and 5), although a small decrease in MCF was still apparent. The restoration of aPTT to baseline values (Figure 2A-C) distinguished aDabi-Fab from PCC and aPCC. In addition, the mean plasma concentration of dabigatran was below the limit of detection (that is, approximately zero) after the addition of both concentrations of aDabi-Fab (Table 1).

## Discussion

This experimental animal trial is the first to show *in vitro* that PCC, aPCC and aDabi-Fab are effective for the reversal of dabigatran-induced coagulopathy in a model of anticoagulation and liver trauma. Coagulation parameters, which were all affected by dabigatran administration, were corrected most effectively by aDabi-Fab, but, this product is yet to be licensed for clinical use. It is therefore of potential clinical importance that this study also suggests that PCC and aPCC could potentially be used for emergency reversal of dabigatran. In contrast, rFVIIa had no significant impact on coagulation after oral DE or intravenous dabigatran administration, despite the use of relatively high-dose rFVIIa (equivalent to 180 μg/kg).

Theoretically, the use of prothrombin (a major component of PCC) may reverse the anticoagulation activity of dabigatran by providing thrombin for the transformation of fibrinogen to fibrin. PCCs and aPCCs are derived from human plasma and most contain the vitamin K-dependent coagulation factors II, VII, IX, and X. PCCs with low levels of factor VII (3-factor PCCs) are commonly used in the USA, but products with higher levels of factor VII (4-factor PCCs) are used more commonly outside the USA. Based on the results of an open-label, phase IIIb randomised controlled trial [18], the first 4-factor PCC (Kcentra, CSL Behring, Germany) has been recently licensed in the US for the urgent reversal of coagulation factor deficiency induced by vitamin K antagonist (for example, warfarin) therapy in adult patients with acute major bleeding [19,20]. Currently, there are two major indications for PCC: rapid reversal of oral anticoagulation (vitamin K antagonists) and deficiency of vitamin K-dependent coagulation factors in life-threatening bleeding. Unlike 4-factor or 3-factor PCCs, aPCC (FEIBA, Baxter, USA) contains activated factors and is indicated for haemophilia A and haemophilia B patients with inhibitors, either to control spontaneous bleeding episodes or for use during surgical interventions [21].

PCC has been successfully used to terminate serious bleeding in pre-clinical studies [22-24]. Evidence from pre-clinical studies also supports a potential role for the use of PCC to reverse the effects of dabigatran [11-13]. In a rabbit model of anticoagulation associated with dabigatran, the use of PCC significantly reduced blood loss in a dose-dependent manner following a standardised kidney incision [11]. Furthermore, in a murine model of intracerebral haemorrhage, animals receiving dabigatran twice daily showed an expansion of intracerebral haematoma on magnetic resonance imaging [13]. The use of PCC (up to 100 U/kg) was associated with dose-dependent prevention of haematoma growth and also reversal of prolonged tail bleeding time. In another murine study, administration of PCC in combination with rFVIIa or aPCC prior to tail tip resection significantly reduced bleeding time but had no significant impact on blood loss, although the dose of PCC used in this study was low (14.3 U/kg) [12].

Few data exist on the use of PCC for the reversal of the anticoagulant effects of dabigatran in humans. In one study, following oral administration of dabigatran to 10 healthy volunteers, the capacity of thrombin generation was evaluated *ex vivo*. The intake of dabigatran was shown to affect the kinetics of thrombin generation, with a prolongation of the lag time and time to peak [14]. The addition of PCC significantly increased the endogenous thrombin potential, although no influence on the lag time was observed. In contrast to this study, in a trial of six healthy volunteers, PCC did not reverse the prolongation of the aPTT resulting from standard oral DE doses of 150 mg twice daily [15].

Although the investigators in the latter study used a different PCC (Cofact; Sanquin Blood Supply, Amsterdam, the Netherlands) to the PCC used in this study (Beriplex P/N 250, CSL Behring GmbH, Marburg, Germany), we have previously shown the two PCCs to have a similar pattern of thrombin generation [25]. Thus, discrepancy between studies regarding the effect of PCC on plasma coagulation assays raises the question of which coagulation test is adequate for monitoring the potential of a PCC to reverse the anticoagulant effects of dabigatran. The reversal of the prolonged PT, but not prolonged aPTT by PCC/aPCC in this study highlights the need for an assay that is sensitive not only to the anticoagulant used, but also to the effects of the reversal agent. In the case of warfarin, the PT (or international normalized ratio) is dependent on the vitamin K-dependent coagulation factors [26] and therefore serves not only as a sensitive measure of the effects of anticoagulation therapy, but also of its reversal by PCCs or aPCC. Conversely, PT is an insensitive measure of the effects of therapeutic doses of dabigatran [26-28]. However, as shown in this study, a high concentration of the drug can prolong the PT, thus enabling the dabigatran-prolonged PT to be reversed by PCC and aPCC. aPTT is more sensitive than PT to therapeutic levels of dabigatran [26,28], but the lack of effects of PCCs and aPCCs on aPTT suggests that this test is not suitable for monitoring reversal with these products. For confirmation of reversal of dabigatran therapy in the presence of trauma-related bleeding, well-designed studies with appropriate (sensitive) coagulation assays are urgently warranted.

In this study, we used thromboelastometry to monitor the effects of dabigatran. In the trauma setting, delays in the detection of coagulopathy may influence outcome; in contrast to the conventional coagulation tests, which are associated with slower turnaround times, thromboelastometry allows rapid assessment of a patient’s coagulopathy. Based on findings from retrospective studies, thromboelastometry appears to be a useful tool to guide PCC therapy in patients with traumatic coagulopathy [29,30]. The EXTEM assay is similar to PT in that it assesses tissue factor-initiated extrinsic coagulation, making it the most suitable thromboelastometric assay for investigating PCC or aPCC reversal of dabigatran. There has been little investigation of the effects of dabigatran on viscoelastic coagulation parameters but, consistent with the present findings, prolonged activated clotting time with rapid thromboelastography was seen in several patients taking dabigatran [31]. We found that PCC, aPCC and aDabi-Fab reversed the anticoagulant effects of dabigatran as shown by improvements in CT and CFT. It is important to note that the decrease in MCF observed after dabigatran infusion was mainly attributable to a decrease in the amount of thrombin available for the conversion of fibrinogen to fibrin, as opposed to insufficient clot substrate (fibrinogen, platelets). Once sufficient thrombin becomes available through addition of PCC, aPCC or aDabi-Fab, transformation of fibrinogen to fibrin is restored and clot formation is no longer impaired. Overall, thromboelastometry may be useful in the detection of coagulopathy associated with dabigatran and in monitoring the effects of reversal therapy.

A drawback of PCC use is the potential risk of thromboembolic complications [24]. We have shown in an experimental animal model of liver injury that high levels (50 U/kg) of PCC may increase the risk for disseminated intravascular coagulation, and this was explained by an imbalance of pro- and anti-coagulant proteins [24]. However, there has been a suggestion that the procoagulant effects of high-dose PCC are reduced in the presence of dabigatran [32]; the safety profile of PCC or aPCC for the reversal of dabigatran remains to be characterised.

rFVIIa is approved to treat haemophilia patients with inhibiting antibodies against coagulation factors VIII or IX [33]. The use of this agent in patients with bleeding trauma who are resistant to conventional haemostatic therapy, even as a last resort, may be considered as surprising given that a large randomised controlled trial (CONTROL) was stopped after an analysis predicted that the likelihood of a successful outcome concerning the primary endpoints (mortality and morbidity) was very low [34]. In the present study, rFVIIa had no effect on reversal of any coagulation parameters. This is consistent with previous data demonstrating lack of effect of rFVIIa on dabigatran-induced bleeding in a murine model of haemorrhagic stroke [13]. These findings may be explained by the mechanism of action of rFVIIa: it influences the kinetics of thrombin generation, but it does not increase the concentration of prothrombin.

The results of this *in vitro* study have shown the specific antidote to dabigatran, aDabi-Fab, to have the greatest impact on all coagulation variables at all measured time points. Dabigatran, even at supratherapeutic concentrations (1,400 to 1,500 ng/mL, compared with the normal therapeutic range in humans of 50 to 250 ng/mL), was completely neutralised after the addition of aDabi-Fab. This is in line with results from a previous rat model of anticoagulation in which a single bolus injection of aDabi-Fab completely reversed the anticoagulant activity of dabigatran. This reversal was maintained for approximately 25 minutes despite continued infusion of dabigatran [16]. The increased potency of aDabi-Fab in comparison with PCC, aPCC and rFVIIa is undoubtedly related to their different mechanisms of action. The aDabi-Fab binds directly to dabigatran in plasma or whole blood and inactivates it, thus all coagulation measurements are returned to baseline. In contrast, PCC, aPCC and rFVIIa do not bind to dabigatran. Instead, these drugs overcome the anticoagulant activity by increasing the availability of substrate for coagulation.

There are some limitations of our study that need to be acknowledged. Haemostatic agents and aDabi-Fab were supplemented *ex vivo*. This approach was taken mainly for ethical reasons: it enabled investigation of a variety of haemostatic agents with a small number of animals. As a consequence, however, the effects of treatment on blood loss could not be measured and it is uncertain to what extent our observations may be predictive of clinical dabigatran reversal. This uncertainty was in part mitigated by the use of intravenous dabigatran in its active form rather than the orally administered pro-drug DE. Thus, possible species-related differences in drug absorption and conversion to active form cannot have influenced the present data. However, there remains a possibility of species differences in aspects such as the tissue factor/rFVIIa complex, and the aPTT/PT responses to dabigatran [35]. Another consideration regarding applicability to the clinical setting is the plasma level of dabigatran. Mean levels at the low dabigatran timepoint were slightly above the normal therapeutic range, with higher values with high dabigatran and post-trauma. Although our study would have been improved by matching more closely the normal therapeutic range of dabigatran levels, it seems reasonable to assume that if reversal is successful at supratherapeutic concentrations, it would also be successful at lower, therapeutic levels. Clinical data from human patients receiving dabigatran therapy are clearly needed to confirm the efficacy, optimal dosing and safety of PCC, aPCC and aDabi-Fab for the reversal of dabigatran.

## Conclusion

In summary, dabigatran-induced coagulopathy was reversed by *ex vivo* administration of two different doses of exogenous PCC and aPCC in this porcine model of anticoagulation and blunt liver injury. In contrast, *ex vivo* rFVIIa had no significant effect on coagulation parameters after administration of dabigatran. Unlike PCC or aPCC, *ex vivo* addition of aDabi-Fab provided complete neutralisation of dabigatran as well as full reversal of its effects. However, aDabi-Fab is still in development and is therefore not yet available for clinical use. Although further data investigating the effects of aDabi-Fab and PCCs are urgently needed, the administration of PCC or aPCC could potentially be considered as a therapeutic option to control life-threatening bleeding among patients under treatment with dabigatran.

## Key messages

● PCC and aPCC effectively reverse dabigatran-induced coagulopathy in an anticoagulation/liver trauma model.

● rFVIIa has no significant effect on coagulation in this setting.

● aDabi-Fab provides the most effective reversal of the anticoagulant effects of dabigatran.

● Thromboelastometry variables may help to guide therapy in patients receiving dabigatran.

● Until aDabi-Fab becomes available, administration of PCC or aPCC might be a reasonable intervention for dabigatran-treated patients with life-threatening bleeding.

## Abbreviations

aDabi-Fab: antibody fragment to dabigatran; ANOVA: analysis of variance; aPCC: activated prothrombin complex concentrate; aPTT: activated partial thromboplastin time; CFT: clot formation time; CT: clotting time; DE: dabigatran etexilate; DOAC: direct oral anticoagulant; ECG: electrocardiography; Hb: haemoglobin; MCF: maximum clot firmness; PCC: prothrombin complex concentrate; PT: prothrombin time; rFVIIa: recombinant activated factor VII.

## Competing interests

OG has received research funding from Novo Nordisk, Biotest, CSL Behring, Nycomed. He has also received honoraria for consultancy and/or travel support from Bayer Healthcare, Boehringer Ingelheim and CSL Behring. RR has received honoraria for lectures and consultancy from CSL Behring and Novo Nordisk. JvR is an employee of Boehringer Ingelheim Pharma GmbH & Co., Germany. HS has received research funding from Boehringer Ingelheim and honoraria for consultancy from Bayer.

## Authors’ contributions

OG conceived and conducted the experimental laboratory work and drafted the manuscript. RR, HS and JvR helped to draft the manuscript. All authors read and approved the final manuscript.
